# Insulin Resistance in Patients With Acromegaly

**DOI:** 10.3389/fendo.2019.00509

**Published:** 2019-07-30

**Authors:** Greisa Vila, Jens Otto L. Jørgensen, Anton Luger, Günter K. Stalla

**Affiliations:** ^1^Division of Endocrinology and Metabolism, Department of Internal Medicine III, Medical University of Vienna, Vienna, Austria; ^2^Department of Endocrinology and Internal Medicine, Aarhus University Hospital, Aarhus, Denmark; ^3^Max Planck Institute of Psychiatry, Munich, Germany

**Keywords:** glucose, insulin, growth hormone, IGF-I, acromegaly complications, diabetes, comorbidities, pathophysiology

## Abstract

Acromegaly is characterized by chronic overproduction of growth hormone (GH) that leads to insulin resistance, glucose intolerance and, ultimately, diabetes. The GH-induced sustained stimulation of lipolysis plays a major role not only in the development of insulin resistance and prediabetes/diabetes, but also in the reduction of lipid accumulation, making acromegaly a unique case of severe insulin resistance in the presence of reduced body fat. In the present review, we elucidate the effects of GH hypersecretion on metabolic organs, describing the pathophysiology of impaired glucose tolerance in acromegaly, as well as the impact of acromegaly-specific therapies on glucose metabolism. In addition, we highlight the role of insulin resistance in the development of acromegaly-associated complications such as hypertension, cardiac disease, sleep apnea, polycystic ovaries, bone disease, and cancer. Taken together, insulin resistance is an important metabolic hallmark of acromegaly, which is strongly related to disease activity, the development of comorbidities, and might even impact the response to drugs used in the treatment of acromegaly.

## Introduction

The main physiological roles of growth hormone (GH) are the regulation of postnatal growth and lipolysis. These actions are highly dependent on the nutritional state, which partitions the metabolic actions of GH (1). GH is released from pituitary somatotroph cells in a pulsatile fashion that is tightly controlled by hormones and nutrients (2–5). GH secretion is enhanced by growth hormone-releasing hormone, fasting, stress, exercise and hypoglycemia, and suppressed by somatostatin, insulin, insuline-like growth factor I (IGF-I), glucose and free fatty acids (1, 3–10).

GH excess in acromegaly results, with few exceptions, from a benign tumor of pituitary somatotroph cells and leads to chronically increased GH concentrations, which do not respond to the classical physiological feed-back inhibition (11). Therefore, acromegaly is characterized by a concomitant increase in both GH and IGF-I production and activity. The main metabolic consequence of acromegaly is insulin resistance, which may progress to diabetes. The underlying pathophysiological mechanisms are increased lipolysis, reduced peripheral glucose utilization and enhanced gluconeogenesis (12). Acromegaly is a unique condition of concomitant increases in GH, IGF-I, and insulin concentrations, where the increase in insulin resistance, paradoxically, is associated with reduced total body fat and even reduced fat accumulation in metabolic organs such as the liver (13, 14).

In the present review, we discuss the mechanisms leading to insulin resistance in patients with acromegaly, the pathophysiological implications of insulin resistance in the comorbidities of acromegaly, as well as the relationship between glucose homeostasis and disease activity in acromegaly.

## Whole-Body Glucose Homeostasis in Acromegaly

### GH and Glucose Homeostasis

The insulin-antagonistic effects of GH were initially described about 85 years ago following the observation that hypophysectomy performed in dogs improved hyperglycemia and experimental diabetes (15). In the second half of the twentieth century, studies performed using pituitary human GH extracts demonstrated direct effects of GH on lipolysis and hyperglycemia (16, 17). It was hypothesized that the GH-induced insulin antagonistic effect is strongly related to the lipolytic effects of GH, as free fatty acids released from fat stores inhibit glucose disposal, resulting in insulin resistance (16, 18). This constitutes a favorable metabolic adaptation to fasting and exercise (where insulin levels and activity are low) by providing lipid utilization at the expense of glucose. By contrast, GH is suppressed postprandially where insulin activity is maximal (16, 17). In addition, GH and insulin pathways have been shown to cross-talk at the postreceptor level in rodent models and *in vitro* (19), but this has not been replicated in human *in vivo* models (20). Since the physiological reciprocal temporal pattern of GH and insulin is abolished in active acromegaly, where the continous GH elevation chronically activates intracellular GH signaling, it remains possible that this could impair insulin signaling, hence causing insulin resistance.

GH signaling in muscle and fat tissues is confirmed already 30 min after a GH surge (21). Intravenous administration of GH in healthy human volunteers leads within 2 h to an increase in free fatty acids together with reduced glucose uptake and oxidation in the muscle in concomitance with increased non-oxidative glucose diposal and increased endogenous glucose production (17, 22). The GH-induced impairment of glucose uptake is causally linked to the concomitant activation of lipolysis, as the administration of the antilipolytic agent acipimox abrogates GH actions on insulin sensitivity (23). It is likely that also the GH-induced stimulation of gluconeogenesis is positively influenced by the increased free fatty acid levels (24).

**Figure 1:**
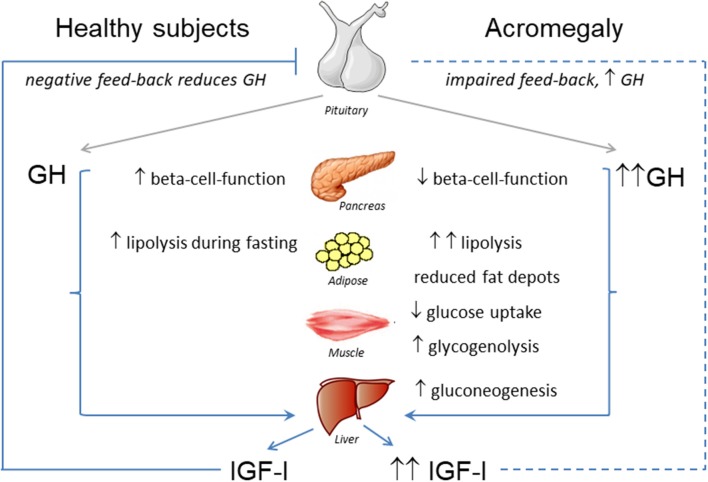
Effects of GH-IGF-I hypersecretion in metabolic organs.

Taken together, GH-induced insulin resistance seems to be mainly the consequence of the increased lipolysis, impaired insulin action in peripheral tissues leading to reduced glucose uptake, and also increased gluconeogenesis in the liver [(17); Figure 1].

### IGF-I and Glucose Homeostasis

Prolonged administration of GH in the context of a positive energy balance leads to production of IGF-I by the liver. IGF-I is a single-chain polypeptide with 50% amino acid sequence homology with insulin, and its synthesis is stimulated not only by GH, but also by insulin (25). Acute increases in IGF-I concentrations exert insulin-like effects on glucose transport and circulating glucose concentrations, also in the absence of the insulin receptor (26). Nevertheless, circulating IGF-I does not cause hypoglycemia, as >90% is bound to specific binding proteins (27). Exogenous administration of IGF-I in high doses, on the other hand, mimicks administration of insulin and therefore acutely lowers plasma glucose levels (28). The same group showed that prolonged administration of IGF-I during several days does not impact circulating glucose concentrations, maybe because the increased IGF-I activity was balanced by a feedback-induced suppression of GH as well as insulin (29). Taken together, IGF-I has insulin-agonistic actions, thereby potentially counteracting the insulin-antagonistic effects of GH. Cross-sectional population studies have shown that both low and high IGF-I are associated with increased insulin resistance, highlighting the complexity of the IGF-I system, which probably reflects that IGF-I is a sex- and age-dependent biomarker of not only GH activity but also nutritional status (30). Circulating IGF-I *per se*, however, probably plays a very minor role in the regulation of glucose homeostasis in acromegaly.

### Glucose Metabolism in Acromegaly

The overall effect of acromegaly on glucose metabolism is mainly determined by the insulin-antagonistic effects of chronically increased GH, which induces hepatic and peripheral insulin resistance as previously mentioned (12, 17, 31, 32). This is followed by a compensatory increase in beta-cell-function, which aims at maintaining euglycemia (33, 34) (Figure 1). Over time, chronic insulin resistance and fatty acid-induced lipotocixity deteriorate beta-cell-function eventually leading to diabetes (33, 34).

An impairment of glucose metabolism is observed in over 50% of patients with newly diagnosed acromegaly (35). Glucose homeostasis is related to disease activity in acromegaly, as higher IGF-I concentrations were found associated to lower insulin sensitivity (36). Nutrient intake and glucose physiologically suppress GH secretion in healthy subjects, but not in patients with acromegaly (37). Indeed, the latter is utilized in the biochemical evaluation of acromegaly disease activity (38).

Surgical cure of acromegaly improves insulin sensitivity and lowers circulating glucose and insulin concentrations (32, 34, 39).

### Body Composition in Acromegaly

GH transgenic mice are resistant to high fat diet induced obesity, exhibiting an accumulation of lean tissue, and no increase in adipose tissue mass (40, 41). Based on the lipolytic effects of growth hormone one would predict a remarkable reduction of total body fat in acromegaly. Indeed, all studies evaluating body composition consistently describe reduced total fat content and also reduced organ-specific fat deposition in patients with acromegaly (13, 14, 42, 43). Nevertheless, one study demonstrated higher intermuscular adipose tissue depots in the presence of lower visceral and subcutaneous fat in patients with acromegaly (44). This is compatible with the observation of increased intramyocellular triglyceride (IMTG) accumulation in healthy subjects after 8 days high dose GH administration (45). Whether IMTG per se contributes to insulin resistance as indicated by other studies not involving GH (46) is uncertain, and it is noteworthy that IMTG also increases following exercise in fit individuals without compromising insulin sensitivity (47).

Ectopic lipids, however, play an important role in the pathophysiology of insulin resistance accompanying obesity in the general population (48). The presence of insulin resistance in the absence of hepatic lipid accumulation in patients with acromegaly is unique, and it is likely that the small increase in intramuscular fat in active acromegaly mainly reflects increased lipid oxidation in muscle. In all instances, biochemical control of acromegaly reverses this picture, increasing total fat mass and reducing lean body mass, while improving insulin sensitivity (13, 42, 43).

## Pathophysiology of Insulin Resistance in Acromegaly

### Fat Metabolism

Lipids constitute the main energy reserves in human physiology, being primarily stored in the adipose tissue as lipid droplets containing triacylglycerides surrounded by a phospholipid monolayer. An acute reduction of circulating free fatty acid levels stimulates GH secretion (10, 49). GH, in turn, stimulates lipolysis in humans leading to increased concentrations of free fatty acids and glycerol (50). Endogenous GH is essential for the increased lipolytic rate found during prolonged fasting, with fasting-induced peaks in endogenous GH secretion being crucial for the increased rate of lipolysis during starvation (51, 52). During the fed state, GH secretion is suppressed and insulin becomes the main regulator of substrate metabolism (10, 49). This feeding-induced shift between insulin and GH in the control of substrate metabolism was already suggested in 1963 by Rabinowitz and Zierler, with GH being responsible for the utilization of endogenous lipids during fasting and stress, thereby sparing glucose and proteins (16).

Acromegaly is associated with increased circulating levels of lipid intermediates, as well as with increased lipid uptake by the muscle, suggesting that fatty acids are a major fuel substrate in these patients (32). This sustained stimulation of lipolysis has three main consequences: (1) further deterioration of insulin sensitivity, (2) impairment of beta-cell function, and (3) reduction of whole-body fat. Surgical cure of acromegaly is followed by a normalization of lipolysis and glucose metabolism (32).

### Beta-Cells

GH stimulation exerts insulinotrophic effects on β-cells *in vitro* (53) and *in vivo*, augmenting glucose-induced insulin secretion without playing a major role in basal insulin secretion (54). The pathophysiology underlying beta-cell-failure in acromegaly seems similar to that observed during the development of type 2 diabetes where insulin resistance leads to a compensatory hyperfunction of beta-cells (33, 34). Insulin resistance and lipotoxicity eventually lead to beta-cell dysfunction with failure to fully counterbalance the increased needs for insulin secretion (33, 34). Several studies have shown an improvement in beta-cell function after normalization of GH concentrations in patients with acromegaly (39, 55). Despite direct effects of GH on glucose metabolism, other hormonal alterations accompanying acromegaly may influence beta-cell function: 1) Acromegaly is associated with increased postprandial glucose-dependent insulinotropic polypeptide (GIP) concentrations, which in turn stimulate insulin secretion and influence postprandial (but not fasting) hyperinsulinemia (56); 2) A cross-sectional study found that beta-cell function strongly and directly correlates with the bone marker osteocalcin, revealing that the bone-beta-cell cross-talk initially described in other populations is also present in patients with acromegaly (57).

### Liver

GH signaling in the liver is essential for the production of IGF-I and for the maintenance of a normal hepatic lipid metabolism (58). GH-induced hepatic IGF-I production depends on the local availability of insulin, with increased hepatic sensitivity to GH in the presence of high portal insulin levels (59). Higher portal insulin concentrations are associated with increased IGF-I concentrations, partially also due to the insulin-induced inhibition of hepatic IGFBP1 production (60). Patients with acromegaly have an increased glucose turnover, as GH increases hepatic glucose production by increasing glycogenolysis (17). Chronic GH administration impairs insulin sensitivity in the liver, thereby reducing the ability of insulin to suppress gluconeogenesis and glucose output (12, 32, 61).

### Muscle

Local GH perfusion in the brachial artery leads to a rapid decrease in muscle glucose uptake and oxidation (17, 62, 63). These rapid GH effects in muscle cells could be either direct or secondary to the increased lipid utilization (17). GH signaling in muscle induces signal transducer and activator of transcription 5 (STAT5) phosphorylation and increased expression of canonical GH-dependent genes including IGF-I and cytokine-inducible SH2-containing protein (17, 21). The molecular mechanisms subserving GH-induced insulin resistance in human subjects remain uncertain. Studies in rodent models show impairment of insulin signaling at the level of phosphoinositide 3-kinase (PI3K) activity (64), but this is not observed in human studies *in vivo* (20, 21, 65, 66).

On the other hand, it has been shown that GH infusion increases lipolysis and suppresses pyruvate dehydrogenase activity, which indicates substrate competition between glucose and lipid intermediates (67) in accordance with the Randle hypothesis (18). However, it is noteworthy that human *in vivo* studies rely mainly on mRNA and protein expression in crude biopsies at specific time points, which may not be able to detect real time changes in complex signaling pathways.

### Adipose Tissue

In general, circulating GH concentrations are inversely correlated to adipose tissue mass in both mice and humans (68). GH directly impairs glucose utilization in 3T3 adipocytes and also impairs insulin signaling in adipose tissue by influencing p85α expression that suppresses PI3K activity (69–72). GH receptor signaling phosphorylates the tyrosine residues on STAT5, leading to STAT5 activation (73). STAT5 mediates the GH-effects on lipolysis by increasing the transcription of several metabolic genes such as peroxisome proliferator-activated receptor (PPAR)-gamma and fatty acid synthase (73, 74). In addition, both STAT5- and mitogen-activated protein kinase (MEK)/extracellular signal-regulated kinase (ERK)-dependent intracellular signaling mediate the effects on GH in suppressing mRNA and protein levels of fat-specific protein 27 (FSP27), a negative regulator of lipolysis (75). The importance of FSP27 supression for GH signaling in adipocytes is highlighted by the fact that FSP27 overexpression fully abrogates the effects of GH on lipolysis and insulin resistance in adipose tissue, mainly by inhibiting PPAR-gamma phosphorylation (75, 76).

Acromegaly is also associated with decreased expression of the insulin-sensitizing adipokine adiponectin, but also with increased circulating concentrations of the proinflammatory adipokine visfatin, which is linked to enhanced inflammation and insulin resistance in many tissues (77–79). In addition, GH excess in acromegaly increases the expression of proinflammatory cytokines within the adipose tissue, which in turn might also contribute to the increased insulin resistance (80). This finding confirms that adipose tissue inflammation can be found also in the absence of increased adipose tissue mass. The functionality rather than the size of adipose tissue mass seems to determine the phenotype, as successful treatment of acromegaly resulted in a reduction of lean body mass and increase of total body fat mass together with improvement of insulin sensitivity and a reduction in proinflammatory cytokines (80).

## Role of Insulin-Resistance in the Development of Acromegaly-Associated Comorbidities

### Diabetes

**Figure 2:**
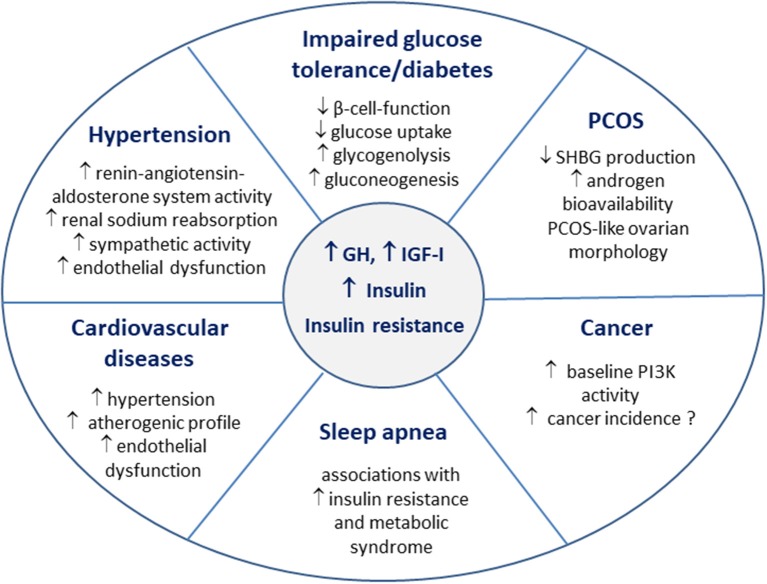
Insulin resistance in the pathophysiology of acromegaly comorbidities.

Diabetes is a late consequence of impaired glucose metabolism in acromegaly, and occurs when the increased beta-cell function fails to compensate for the chronically increased insulin resistance (33, 34) (Figure 2). The prevalence of diabetes in patients with acromegaly is 20–35% at initial disease diagnosis (81, 82). The frequency of diabetes is related to disease control in acromegaly, and IGF-I concentrations are higher in patients with diabetes, when compared to patients with impaired glucose tolerance or normal glucose metabolism (81, 83). Age and positive family history for diabetes were found to be independently associated with impairment of glucose metabolism in acromegaly (35).

### Hypertension

Insulin resistance is thought to be one of the main factors contributing to hypertension in the general population (84, 85). In acromegaly, hypertension is the most prevalent cardiovascular comorbidity, described in up to 60% of published acromegaly cohorts (86–90). The contribution of insulin resistance in the development of hypertension was studied in GH transgenic mice, in which increased systolic blood pressure appears at the age of 3–6 months and the prevalence of hypertension increases with age (91). In this model hyperinsulinemia develops very early, and unlike hypertension, even improves with age. The authors conclude that hyperinsulinemia might play a role in the pathophysiology of hypertension, but is not mandatory for the maintenance of hypertension (91).

Hyperinsulinemia may lead to increased sodium absorption in the kidney by activating the renin-angiotensin-aldosterone system, thereby also increasing the circulating plasma volume (92). In addition, both insulin and growth hormone increase sympathetic nervous activity (93). In accordance with this, patients with acromegaly lack the nocturnal fall in norepinephrine and blood pressure levels (94).

Insulin resistance and hyperinsulinemia impair also endothelium-dependent vasodilatation and increase oxidative stress in endothelial cells (95, 96). Indeed, flow-mediated vasodilatation, a functional test used for detecting endothelial dysfunction was lower in patients with acromegaly when compared to age- and gender-matched controls (97). Transsphenoidal surgery led to an increase/normalization of flow-mediated vasodilatation only in subjects that showed significant improvements in glucose, insulin concentrations and insulin resistance (97). The authors conclude that endothelial function in patients with acromegaly strongly relates to insulin resistance and does not always change following rapid improvement in GH and IGF-I concentrations (97). Interestingly intima media thickness of common carotid arteries, but not the prevalence of atheroclerotic plaques, is increased in patients with acromegaly (98). Taken together, insulin resistance is one of the main pathophysiological mechanisms contributing to the development of hypertension in acromegaly (99). In routine clinical practice, patients with acromegaly and impaired glucose tolerance/diabetes have higher blood pressure values than patients with acromegaly and normal glucose tolerance (100). In larger populations, the presence of diabetes in patients with acromegaly is associated with a significantly increased prevalence of hypertension (101).

### Cardiovascular Disease

Insulin resistance is an independent risk factor for cardiovascular disease in the general population mediating the association between hyperglycemia and cardiovascular risk (102–104). Impaired glucose tolerance directly correlates to the severity of acromegalic cardiomyopathy (100). Patients with acromegaly and diabetes show an increased prevalence of cardiovascular diseases (101). GH and IGF-I excess lead to morphological heart changes (86, 105). Acromegalic cardiomyopathy displays similar features as diabetic cardiomyopathy, and the main morphological difference between the two is the lack of intracardiac lipid accumulation in acromegaly (14, 99). The coexistence of hypertension further aggravates cardiomyopathy in acromegalic patients (105, 106). Although there is no evidence for a direct effect of insulin resistance on heart morphology and dysfunction in acromegaly, the atherogenic properties of insulin resistance are thought to contribute to the pathophysiology of cardiovascular diseases [(99); Figure 2].

### Obstructive Sleep Apnea

Obstructive sleep apnea is the most common sleep disorder in patients with acromegaly, and also specifically associated with increased mortality (89, 90, 92, 99). The prevalence of sleep apnea in acromegaly ranges from 45 to 80% and does not reliably relate to disease activity, with conflicting studies on its reversibility following successful treatment of acromegaly (92, 107).

The pathophysiology of sleep apnea in acromegaly is mainly based on soft tissue thickening of bronchial, pharyngeal, and laryngeal mucosa, as well as craniofacial morphological changes (92). Nevertheless, several studies support a bidirectional link between sleep apnea and insulin resistance/metabolic syndrome in the general population (108, 109).

### Polycystic Ovary Syndrome (PCOS)

Both GH and IGF-I are known to affect ovarian function and morphology (110). Hypogonadism and menstrual irregularities are often reported in women with acromegaly, as volume effects of the pituitary adenoma and GH excess may lead to hypogonadotropic hypogonadism (111). Insulin directly modulates steroidogenesis in ovarian cells, and insulin resistance plays an important role in the pathogenesis of PCOS (112). GH modulates ovarian function both directly and via IGF-I (110). Hence, not only GH/IGF-I, but also hyperinsulinemia and insulin resistance accompanying acromegaly seem to be involved in the pathogenesis of a PCOS-like ovarian morphology, which was described in 50% of women with acromegaly (113). Although androgen levels are not generally increased in women with acromegaly, the circulating levels of sex hormone binding globulin (SHBG) were found to be low in a retrospective analysis (114). Insulin resistance is associated with decreased SHBG production in the liver, which in turn leads to increased androgen bioavailability, and this along with a direct stimulatory effect of GH on hair growth seems to be one of the reasons underlying the increased prevalence of hirsutism in women with acromegaly (113, 115). Therefore, the clinical phenotype of PCOS is determined not only by the severity of hyperandrogenism, but also by the degree of insulin resistance/hyperinsulinemia (Figure 2).

### Cancer

High IGF-I levels are associated with an increased rate of malignancies in the general population (116, 117). Elevated GH and IGF-I concentrations might promote the development and progression of malignancies in patients with acromegaly but this remains a controversial topic (90). A recent meta-analysis revealed a moderate increased cancer risk, but this is mainly observed in single center studies (118). While older retrospective studies showed increased mortality due to cancer in acromegaly, the more recently published reports on acromegaly cohorts with normalized GH and IGF-I levels seem to indicate that the cancer mortality is comparable to the one observed in the general population (117, 119).

Insulin resistance and metabolic syndrome are also associated with an increased incidence of cancer (120). Indeed, the PI3K pathway, a common target of GH and insulin signaling, remains an important treatment target in malignancies (121). Other mitogenic pathways induced by both GH and insulin are MAPK/ERK and Ras-like GTPases (117). To date, there is no direct evidence on an additive effect of insulin resistance in the development of cancer in patients with acromegaly.

### Bone Disease

Patients with acromegaly display a significant impairment of bone microarchitecture, increased bone formation and resorption, as well as an increased incidence of fractures (122–124). The frequency of fractures in patients with acromegaly relates to disease activity, male gender and concomitant hypogonadotropic hypogonadism (123). Importantly, the prevalence of diabetes is higher in acromegalic patients experiencing fractures (125). Diabetes is associated with an increased fracture risk also after biochemical control of acromegaly (125). To date the pathophysiological evidence linking insulin resistance with bone diseases is scarce, but cohort studies show impaired bone turnover, increased incidence of osteoporosis and fracture risk in non-acromegalic patients with diabetes (126, 127). GH and IGF-1 increase bone turnover and acromegaly is associated with distinct alterations in bone compartments showing lower trabecular bone quantitative parameters, while cortical bone density seems better preserved and found decreased only in patients with vertebral fractures (128). In contrast, diabetes is associated with increased cortical porosity and thinning by trabecularization of the endosteal part of cortical bone (129). So it appears that diabetes and acromegaly affect bone morphology differently. To date it is not clear whether insulin resistance plays a role in the association between fracture risk and diabetes in patients with acromegaly; or whether this is a simple marker of disease severity and thereby associated with other acromegaly complications.

## Insulin Resistance in Relationship to Disease Activity and Therapy of Acromegaly

Biomarkers of glucose metabolism strongly relate to disease activity in patients with acromegaly, where IGF-I serves as a biomarker of overall disease control in acromegaly (35, 36). The impaired glucose metabolism often improves following successful pituitary surgery, and patients in remission have less prevalent diabetes than patients with persistent active disease necessitating medical therapy (32, 34, 39, 81). One of the main factors determining the normalization of glucose metabolism after surgical cure of acromegaly is the beta-cell state. Patients with preserved beta-cell function achieve a normalization of glucose tolerance after surgery, while impaired beta-cell function leads to persistently abnormal glucose metabolism also after successful surgery (55).

The relationship between parameters of glucose homeostasis and drugs used for treating acromegaly has been subject to extensive studies, as recently reviewed (130). A recent report compared the effect of the three main treatment modalities on glucose metabolism in patients with biochemically controlled acromegaly, finding out that plasma glucose decreases after successful surgery and after pegvisomant therapy, but increases in patients using first-generation somatostatin analogs (SSA) (131). First-generation somatostatin analogs (SSA) control GH secretion and IGF-I production, thereby lowering disease activity and improving insulin sensitivity in acromegaly. In parallel, however, they suppress secretion of insulin as well as of gastric and gut peptides, so their overall effect on glucose hoemostasis is not straightforward, but marked deterioration of glucose metabolism is rarely encountered (130, 132). Colao et al. described that the effects of SSAs on glucose metabolism depend on the status of glucose impairment before starting the therapy: SSA may increase plasma glucose levels in patients with normal or impaired glucose tolerance, and this effect was abolished after adding metformin (132). In patients with diabetes, both impairment and improvement of glucose tolerance were observed, and some cases needed optimization of diabetes therapy, but all patients had HbA1c < 6.5% at the end of the study (132). A recent meta-analysis including 47 studies on this topic found a high heterogeneity in fasting glucose and HbA1c outcomes, revealing a significant HbA1c increase over time (133). They describe a marginal and non-significant increase in fasting glucose, which became significant only in the subgroup of patients receiving SSA as second-line therapy, while glucose 2 h after OGTT significantly increased (133). In addition, they observed an improvement in insulin resistance and beta-cell function (133).

The multireceptor-ligand pasireotide more strongly suppresses insulin secretion and gut hormones and therefore hyperglycemia is observed in more than half of the patients (130, 134, 135). In healthy volunteers, pasireotide decreases insulin secretion and the incretin effect, but does not impact insulin sensitivity (135). In patients with acromegaly, improvement of disease control under pasireotide increases insulin sensitivity, but the concomitant impairment of beta-cell function is the main player determining the deterioration of glucose metabolism (130). In 13.2% of patients receiving pasireotide, treatment was withdrawn due to severe hyperglycemia (136).

The growth hormone antagonist pegvisomant was the first acromegaly medication to show a significant improvement in glucose metabolism with overnight reductions in endogenous glucose production and free fatty acid concentrations (137–139). Pegvisomant ameliorates all aspects of glucose metabolism and reduces the need for antidiabetic medications (101, 130). Therefore, pegvisomant is an attractive option in acromegalic patients with poorly controlled diabetes, and its dose requirements also depend on the severity of diabetes (140). The positive effects of pegvisomant on glucose metabolism are preserved when it is combined with an SSA (141–143).

Table 1 summarizes the effects of acromegaly-specific therapies on glucose metabolism. The relationship between dopamine agonist therapy and glucose metabolism has been subject to only a few reports, showing a reduction in basal and stimulated insulin levels (130, 144). Nevertheless, the impact of dopamine agonists on glucose metabolism was extensively studied in patients with prolactinomas, confirming their positive effect in reducing insulin resistance and ameliorating beta-cell function (145).

**Table 1:** Glucose homeostasis in relation to acromegaly treatment options.

	**Fasting glucose**	**Glucose 2 h after OGTT**	**Beta-cell function**	**Insulin resistance**	**HbA1c**
Surgery (32, 39, 55, 131)	↓	↓	Improves	↓	↓
Dopamine agonists (130, 144, 145)	↔	↓	Improves	↓	↓
First-generation SSAs (131–133, 146, 147)	↔ / ↑	↑	Deteriorates/improves	↔ / ↓	↔ / ↑
Pegvisomant (101, 131, 137–139, 148, 149)	↓	↓	Improves	↓	↓
Pasireotide (134–136, 150)	↑	↑	Deteriorates	↔ / ↓	↑
SSAs + Pegvisomant (143, 151)	Positive effects of GH/IGF-I reduction counteract the SSA-induced impairment of beta-cell function, no change in HbA1c				
Pegvisomant + Pasireotide (142)	Risk of hyperglycemia is inversely related to insulin secretion at baseline				

*Arrows show increase (↑) or decrease (↓). OGTT, oral glucose tolerance test; SSA, First-generation somatostatin analogs*.

## Summary

Insulin resistance is an important metabolic hallmark of acromegaly caused mainly by the insulin-antagonizing effects of GH in general and the lipolytic effects of GH in particular. The degree of impairment of glucose metabolism is positively related to disease activity in acromegaly and is usually reversed after acromegaly treatment. Insulin resistance plays an important role in the development of acromegaly-specific comorbidities and further studies are needed for elucidating the role of drugs that improve insulin resistance on long-term patient outcomes in acromegaly.

## Author Contributions

GV wrote the initial version of the manuscript. JJ, AL, and GS reviewed the literature and critically revised the manuscript.

### Conflict of Interest Statement

GV has received lecture fees from IPSEN, Novartis, and Pfizer and serves on advisory boards for Pfizer and Novartis. JJ has received lecture fees and reserach grants from IPSEN, Novartis, and Pfizer and serves on advisory boards for Pfizer and IPSEN. AL has received honoraria for lectures from Ipsen, Novartis and Pfizer, participation in advisory boards from Ionis, Ipsen, Novartis, Pfizer. GS has received consultancy fees and/or reimbursements of delegate fees for conferences/educational events and/or travel expenses and/or funding for research projects from Pfizer, Ipsen, Lilly, Shire, Novartis, Sandoz, NovoNordisk, and HRA.

## Abbreviations

ERK: extracellular signal-regulated kinase
FSP27: fat-specific protein 27
GH: growth hormone
IGF-I: insuline-like growth factor I
IMTG: intramyocellular triglyceride
MEK: mitogen-activated protein kinase
PCOS: polycystic ovary syndrome
PI3K: phosphoinositide 3-kinase
PPAR: peroxisome proliferator-activated receptor
SHBG: sex hormone binding globulin
SSA: somatostatin analog
STAT5: Signal transducer and activator of transcription 5.
